# Challenging the citrate challenge: why we need global criteria to define citrate accumulation

**DOI:** 10.1186/s13054-026-05941-x

**Published:** 2026-03-10

**Authors:** Mattia M. Müller, Sascha David

**Affiliations:** 1 Institute of Intensive Care Medicine, University Hospital Zurich and University of Zurich, Zurich, Switzerland; 2 Department of Nephrology and Hypertension, Medical School Hannover, Hannover, Germany

## Dear Editor,

In a recent editorial by our esteemed colleagues Bidar, Khadzhynov, and Rimmelé entitled “The citrate challenge: mitigating the risk of citrate accumulation in high-risk patients” [1], the authors present an algorithm for stratifying patients at risk of citrate accumulation (CA) during continuous kidney replacement therapy (CKRT) with regional citrate anticoagulation (RCA).

While RCA is recommended as the preferred anticoagulant strategy during CKRT by current guidelines, its use in patients at risk of impaired citrate metabolism remains a matter of ongoing debate [2]. While missed or inadequately managed CA has the potential to adversely affect patient outcomes, its overdiagnosis and unnecessary switching to alternative anticoagulation strategies may expose patients to an increased risk of bleeding complications or recurrent filter clotting. Providing practical guidance for the identification of CA is an important and unmet medical need. However, several considerations should be taken into account when implementing such strategies in clinical practice.

### Definition of citrate accumulation

To date, there is no consensus definition for the diagnosis of CA. Meier-Kriesche was among the first to propose the use of the total-to-ionised calcium ratio (tCa/iCa) [3]. However, reliance on tCa/iCa alone has been challenged due to concerns about potential overestimation of CA risk, and several alternative definitions have emerged (Table 1) [4]. Importantly, only a few of these definitions have been evaluated for their association with patient-centred outcomes, and their clinical relevance therefore remains uncertain [5, 6].

### Relevance of vasopressors as a screening tool

High vasopressor doses have been proposed by Bidar and others as a potential screening tool for identifying patients at risk of CA [1, 7]. In a recent study conducted by our own group at the University Hospital Zurich, Switzerland, we found that the vasoactive inotropic score, an aggregate measure of vasoactive and inotropic support, showed poor predictive capability (area under the ROC curve 0.58) in a high-risk population of 911 patients (~ 50% with liver dysfunction) [6]. These findings suggest that single use of “vasopressor requirements” remains questionable as a primary screening tool for CA. Moreover, such pre-screening strategies may generally fail to identify a proportion of at-risk patients and therefore should not replace systematic monitoring using validated parameters of CA such as tCa/iCa.

### Identification of reliable predictors

Several attempts have been made to identify a single reliable and clinically accessible biomarkers for the early detection of CA. In an older study, Khadzhynov and colleagues identified a lactate threshold of 2.39 mmol/L in a cohort of 1,070 patients as an optimal cut-off for distinguishing patients at risk [8]. A similar lactate threshold of 2.6 mmol/L was observed in our Zurich cohort [6]. However, in both studies these thresholds were characterised by a low positive predictive value, raising concerns about potential overestimation of patients considered “at risk”.

**Fig. 1:**
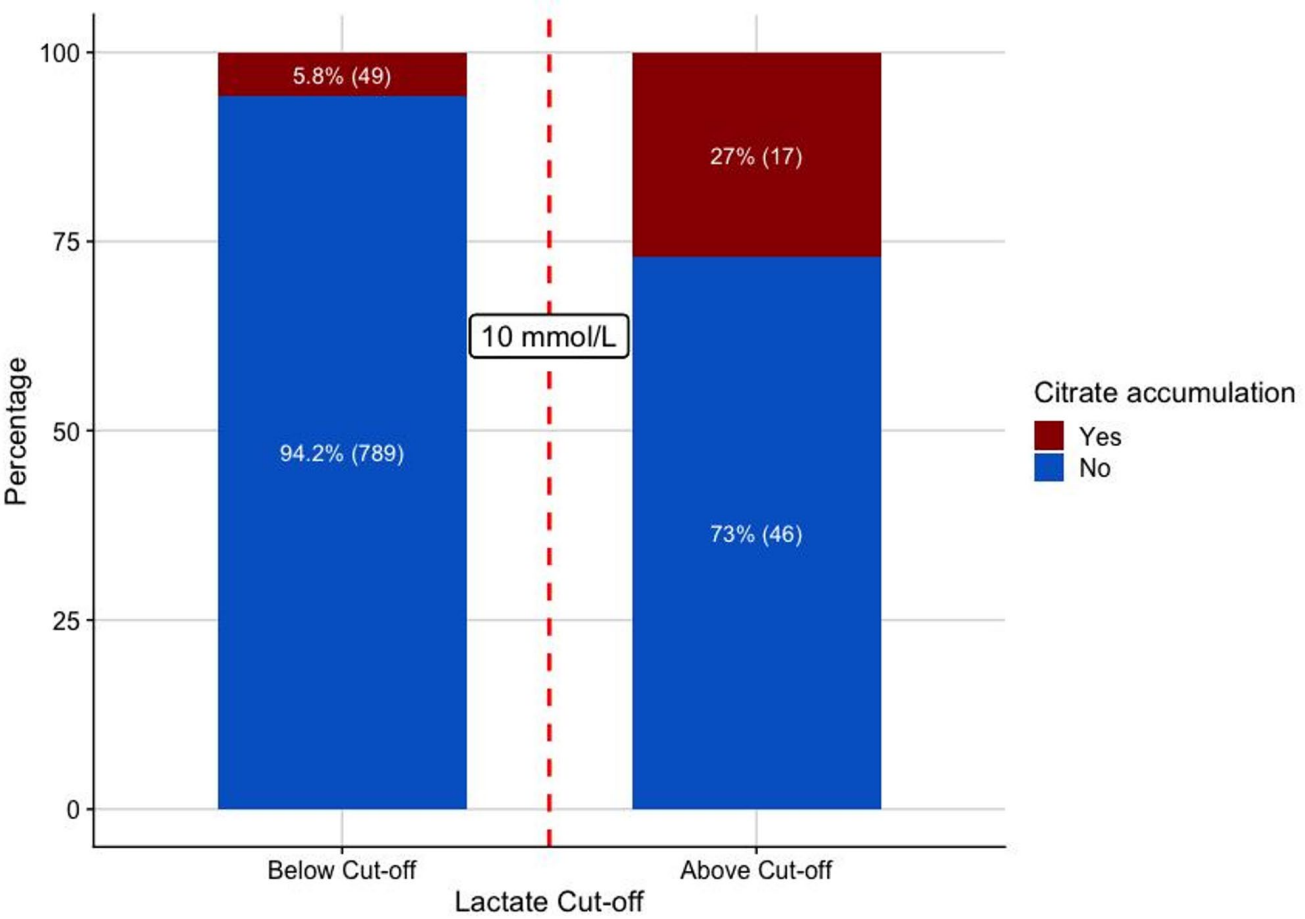
Proportion of patients who developed citrate accumulation (red) within 6 h after the start of CKRT, stratified by lactate levels above or below 10 mmol/L measured prior to CKRT initiation

In their editorial, Bidar and colleagues propose a much higher lactate level > 10 mmol/L or metabolic acidosis with pH < 7.15 as indicators of immediate risk of CA. These thresholds originate from an analysis designed to differentiate patients who developed CA within the first 6 h after initiation of CKRT from those who developed it between 6 and 24 h in a cohort of 69 individuals [9]. Importantly, these thresholds have not undergone internal or external validation for discriminating between patients with and without CA, and therefore their broader applicability remains uncertain. Reevaluating our own recent study, 63 of 911 patients had initial lactate levels of more than 10 mmol/L. Interestingly, 49 out of 66 patients who developed citrate accumulation within the first 6 h after onset of CKRT would not have been detected using this threshold. On the other hand, 73% of these patients with such massively elevated serum lactate did not develop CA in the next 6 h at all (Fig. 1). This suggests that more elaborated models, together with appropriate validation, might be required for accurate risk estimation of CA.

### Management

Once the probability of CA is deemed high, appropriate measures must be implemented to prevent potential harm. Bidar and colleagues propose the initiation of a low-citrate protocol, followed by close monitoring for the development of CA [1]. Transition to an alternative anticoagulation strategy is recommended if CA occurs despite these measures. A similar strategy was reported by Bachmann et al., who demonstrated that introducing a protocol with a reduced blood-to-dialysate flow ratio in high-risk patients allowed continuation of RCA while maintaining a stable incidence of CA [7]. Compared with the preceding period, during which high-risk patients were treated without RCA, this approach was associated with prolonged filter lifespan but also an elevated incidence of metabolic acidosis.

This suggests that it may be safe to continue RCA with adjusted setting (i.e. lower citrate delivery) even in high-risk individuals, but that this approach may simultaneously limit the ability to adequately correct metabolic acidosis. Clinicians should be aware of this limitation, particularly in patients who require rapid correction of severe metabolic disturbances due to their underlying acute condition.

### Need for global criteria

While clinically applicable algorithms for identifying patients at risk of CA are both crucial and highly valuable, further refinement and validation is required. Therefore, we suggest the following steps as necessary to reach global criteria for the identification and management of patients at risk of CA.

1. **Establishing a global consensus definition** of CA with demonstrated clinical relevance.
2. **Identifying and validating reliable criteria** for the early detection of CA according to this definition.
3. **Developing and validating effective management strategies** that are broadly applicable, including in centres with limited experience in managing CA.

Adopting standardised, globally applicable, and validated criteria for identifying CA will mitigate the risk of CA and improve the safety of critically ill patients requiring CKRT.

**Table 1:** Selection of major studies on citrate accumulation and definitions used

Author	Publication year	Number of patients	Criteria of citrate accumulation	Ref.
Meier-Kriesche	2001	161	tCa/iCa ≥ 2.5	[3]
Khadzhynov	2014	1070	iCa < 1.1 mmol/L AND ≥ 2 of the following criteria: - tCa/iCa increased - pH < 7.2 and/or base excess < -5 - anion gap > 11 OR calcium substitution rate > 3 mmol/L	[10]
Link	2012	208	tCa/iCa ≥ 2.4	[5]
Bachmann	2025	467	tCa/iCa > 2.5 AND pH < 7.35 AND increased calcium substitution rate	[7]
Bidar	2025	2080	tCa/iCa > 2.3 AND iCa < 1.1 mmol/L AND ≥ 1 of the following criteria: - pH < 7.2 and/or base excess < -5 - anion gap > 12	[9]
Müller	2025	911	albumin-corrected tCa/iCa ≥ 2.5	[6]

tCa/iCa = Total-to-ionised calcium ratio, iCa = ionised calcium, Ref. = references

## Data Availability

The datasets used and/or analysed during the current study are available from the corresponding author on reasonable request.

## Abbreviations

AUC: Area under the ROC curve
CKRT: Continuous kidney replacement therapy
CVVHD: Continuous venovenous hemodialysis
RCA: Regional citrate anticoagulation
tCa/iCa: Total-to-ionised calcium ratio
VIS: Vasoactive inotropic score
